# Positive Effects of Tomato Paste on Vascular Function After a Fat Meal in Male Healthy Subjects

**DOI:** 10.3390/nu10091310

**Published:** 2018-09-15

**Authors:** Andrea Dalbeni, Davide Treggiari, Angela Tagetti, Michele Bevilaqua, Sara Bonafini, Martina Montagnana, Giuliana Scaturro, Pietro Minuz, Cristiano Fava

**Affiliations:** 1 Department of Medicine, General Medicine & Hypertension Unit, University of Verona, Piazzale LA Scuro 10, 37134 Verona, Italy; angela.tagetti@libero.it (A.T.); bevilacqua.michele@yahoo.com (M.B.); sara.bonafini@univr.it (S.B.); Giuliana.scaturro@gmail.com (G.S.); pietro.minuz@univr.it (P.M.); cristiano.fava@univr.it (C.F.); 2 Department of Biotechnology, University of Verona, 37134 Verona, Italy; davide.treggiari@univr.it; 3 Department of Neurological, Biomedical and Movement Sciences, Laboratory of Clinical Biochemistry, University of Verona, 37134 Verona, Italy; martina.montagnana@univr.it

**Keywords:** Tomato, endothelium, arterial stiffness, stiffness index, fat meal, blood pressure

## Abstract

Tomato consumption has been recently associated with a reduced incidence of cardiovascular disease (CVD). The aim of this study was to test whether a seven-day period of tomato paste purèe (tomato paste, TP) supplementation could improve some haemodynamic parameters in healthy volunteers before and after a standardized fat meal (FM). Methods and results: Nineteen healthy male volunteers participated in a randomized, single-blind (operator) crossover study. Participants maintained low fiber diets (LFD) during the study periods. They were randomized either to a LFD and TP arm (80 g of TP/day) for seven-days, or to a control arm (LFD-only) with a two-week washout period. Flow Mediated Dilatation and other morpho-functional vascular indices were measured by ultrasound. Stiffness Index and Reflection Index were estimated by digital photo-plethysmography. All these parameters were measured one h before and two and 3.5 h after the FM. The difference in Stiffness Index was increased in the LFD and TP + FM-arm, as compared to the LFD-only + FM arm at both two and 3.5 h points. After the FM, in both arms, at two h, we observed a reduction in the Reflection Index and an increase in heart rate. Interestingly, only in the LFD and TP + FM-arm, some haemodynamic changes were detectable at two h; notably, there was an increase in brachial artery diameter and a reduction in diastolic blood pressure (BP). Conclusions: TP has no effect on Flow Mediated Dilatation but acutely modifies some haemodynamic parameters triggered by FM, suggesting possible haemodynamic beneficial effects in people consuming tomatoes.

## 1. Introduction

Tomato is between the main components of the Mediterranean diet and its consumption has been associated with a reduced incidence of cardiovascular disease (CVD) [1]. Tomatoes, and many tomato products, contain potassium, folate, vitamin A, C, E, and other abundant pigmented phytochemicals such as carotenoids, polyphenols, and flavonoids. In particular, both vitamins and carotenoids (α-, β-carotene, lutein, and lycopene) are known to have antioxidant properties and promote cardiovascular (CV) health [2]. Several epidemiological studies [3,4,5,6,7], but not all [8,9], suggest that a high dietary intake of tomato or lycopene and a high plasma level of lycopene are associated with a lower risk of CVD. A recent meta-analysis indicates that an increased intake of tomato and/or lycopene could have a beneficial effect on CV risk factors, including blood lipids, blood pressure, and endothelial function [10].

Nonetheless, it is not clear if lycopene and other antioxidants, present in tomato, are the main mediators of these effects, and if the beneficial effect is mediated by antioxidant, anti-inflammatory, and/or vasoactive effects of these substances. We have recently shown that lycopene can influence the migration of endothelial cells, induced by increasing nitric oxide (NO) bioavailability as stimulated by vascular endothelial growth factor A (VEGF-A) [11], suggest that a pivotal role of NO in this process. 

NO is a vasodilatory substance secreted by the endothelium, which provokes vasodilatation and other beneficial effects [12]. A healthy endothelium can produce more NO and other vasodilatory substances (prostacyclin, epoxyeicosatrienoic acids, etc.) that modulate vascular reactivity. Flow mediated dilatation is an ultrasound-based method to detect the capacity of the endothelium to dilate after increasing shear stress, which has been demonstrated to be at least partially mediated by nitric oxide (NO) [13]. However, apart from flow mediated dilatation, even digital photoplethysmography, which is mainly used with the aim to measure vascular elasticity, when detecting modifications that occur acutely, could be seen as a proxy for the liberation of vasoactive substances, rather than long-term matrix remodeling [14]. Another possibility is that the beneficial effects, at least acutely, could also be seen when a negative stimulus, such as a fat meal, is applied to the endothelium [15]. 

Thus, we hypothesized that short-term consumption of tomato paste (TP) improves vascular function parameters either before or after consuming a standard high-fat meal. The aim of our study was to assess whether one week of supplementation of TP can ameliorate vascular function, before or after a FM, in a sample of male human volunteers randomized in a crossover dietary intervention study.

## 2. Methods 

Most of the volunteers involved in this observational study were students at Verona University. No compensation was given to the volunteers. The exclusion criteria were: Female sex, age < 18 or > 38 years, previous cardio and/or cerebrovascular disease, a clinical diagnosis of hypertension (BP > 140/90 mmHg on several occasions and/or the use of antihypertensive treatment), current smoking, diabetes mellitus or impaired fasting glucose (glucose ≥ 110 mg/dL), hypercholesterolemia (total cholesterol ≥240 mg/dL or anti-lipemic medication use), BMI (Body Mass Index) > 27 or < 19 kg/m^2^, chronic renal disease (serum creatinine ≥ 1.5 mg/dL, chronic inflammatory or neoplastic diseases, use of antioxidant drugs/supplements or nonsteroidal inflammatory drugs (NSAIDs) or COX-2 inhibitors (COXIBs), and food allergy to tomato or other components of the high fat diet. Eligibility criteria were verified by the physicians involved in the study. 

### 2.1. Study Design 

The study was conducted according to an operator-blind cross-over design between October 2013 and December 2015. The administration of a concentration of tomato purèe (TP) began one week before the day of the hemodynamic study (80 g per day of TP, distributed during all the day according to participant’s choice) after a week of low fibers diet (LFD, see flow-diagram). Between the first and the second phase of the study, two weeks of wash-out from any type of food containing tomato was requested. The primary endpoint of the study was the effect of TP on vascular function and in particular on Flow Mediated Dilatation other than on systemic and segmental arterial stiffness. Secondary endpoints were significant changes in the other parameters associated with metabolic syndrome (MetS), according to the AHA/NHLBI (American Heart Association/National Heart, Lung and Blood Institute) criteria such as the HOMA-IR (Homeostasis Model Assessment of Insulin Resistance) index, glucose, cholesterol, and triglycerides. The study was approved by the Ethical Committee of the University Hospital of Verona, and written informed consent was obtained from each participant. The trial was registered in the ClinicalTrials.gov database with the identification code NCT01968369 (16 October 2013). 

### 2.2. Randomization and TOMATO Allocation

The randomization sequence was obtained by a computer-generated random-number list with 2 blocks. A medical doctor who was not otherwise involved in the study, kept the randomization list until the trial was completed. 

Tomato paste (Berni**®**; 100 g of product: protein 1.3 g, carbohydrates 1.7 g, dietary fiber 1.8 g, sodium 0.1 g) was distributed to all participants to be consumed during the day (80 g per 7 days = total load 560 g of TP). During the week before the study, all the subjects were requested to maintain an LFD (to standardize the consumption of lycopene and other antioxidants contained in fruits and vegetables) and only subjects randomized to TP received a daily dose of 80 mg of TP. Healthy volunteers in the LFD-only arm were strictly forbidden to consume tomato in the week before the hemodynamic study.

The day of the hemodynamic study, fasting subjects were evaluated before and after an Italian FM (pasta (50 g) with Parmesan (20 g) and oil (40 g), ham (30 g) and cheese (100 g), potatoes (180 g), and bread (30 g); total calories: 1218 Kcal (14% proteins, 57% total fat, 29% carbohydrates) to be consumed in 30 min. Subjects who were assigned to consume TP since the week before received another dose of TP (80 g) at the same time of the FM. Patient compliance was assessed by interview and by checking the remaining TP on the returned TP bowls. 

### 2.3. Assessments

Each participant was evaluated by the same operator 6 times throughout the study (1 h before the fatty meal consumed at lunch, at 2 and 3.5 h after lunch) once when assigned to the LFD and TP arm, and once when assigned to the LFD-only arm, as shown in the flow diagram. Before the randomization, a questionnaire was administered dealing with the medical history, family history, physiological and pathological information, use of drugs, and smoking. Then, the participants underwent a physical examination. If included, healthy volunteers were advised not to engage in strenuous exercise during the study period (only walking was allowed) and to avoid consuming caffeine-containing beverages within 12 h of the vascular studies. 

During each visit, blood pressure (BP) was measured with a semiautomatic oscillometric device (TM-2501, A and D instruments Ltd., Abingdon, Oxford, UK) 2 times and 2 min apart with the patient lying supine for at least 10 min before the first measurement in a room with a controlled temperature (22–24 °C). Body weight, height, and waist circumference were measured with the volunteers wearing light clothes. A collection of venous blood (in anticoagulated test tubes with ethylendiaminetetracetic acid, ETDA,) and an extemporary urine samples were provided. Urine was stored at −20 °C until use and then thawed at 4 °C and centrifuged at 2400 g for 10 min. Plasma and/or serum were frozen at −20 °C.

Endothelial function was assessed by ultrasound (LogiQ P5 pro, GE Healthcare, Indianapolis, IN, USA) using a high-resolution probe 5–13 MHz with an axial resolution of 0.01 mm using the Flow Mediated Dilatation technique with the upper cuff according to international guidelines [13]. A mechanical arm to fix the ultrasound probe was used. The analysis of images was performed with the aid of a dedicated hardware based on a robust edge detection algorithm that can accurately measure the arterial diameters and the percentage of variation of the diameter after cuff deflation (Multimedia Video Engine II (MVE2) DSP Lab., Pisa CNR, Italy) [16,17]. Common carotid artery distensibility (DC) was calculated as: DC = ∆A/(A × ∆P), where A is the diastolic lumen area, ∆A is the stroke change in lumen area, and ∆P is brachial pulse pressure (PP). Changes in diameters were detected using ultrasound B-mode image sequences of the right and left common carotid arteries, which were acquired at different steps and analyzed by the above mentioned automatic system [18].

Stiffness Index and Reflection Index were estimated by the Digital Volume Pulse (DVP) method and were obtained with the digital photoplethysmography PulseTracePT1000 (MicroMedical Ltd., Gillingham, Kent, UK) [14]. Three measurements were performed and averaged. 

Carotid intima-media thickness (IMT) measurements were performed with the same ultrasound device for Flow Mediated Dilatation. IMT was measured at the level of the distal common carotid artery [19], and presented as the average between the right and left common carotid artery. 

The University Medical Research Laboratory (LURM) of the Integrated University Hospital (AOUI) of Verona measured urinary nitrates using an immuno-enzymatic colorimetric method through a specific laboratory kit (Caymann Chemical Nitrate/Nitrite Assay Kit). The assay was performed according to the procedure recommended by the manufacturer. The intra-inter assay coefficient of variation was 2.7% and 3.4%, respectively. The results were expressed as nitrate/creatinine ratio (µM/µmoL). The blood for laboratory testing was drawn in evacuated blood collection tubes containing lithium-heparin (Terumo Europe N.V., Leuven, Belgium) and kept frozen at −80 °C until measurement. Total cholesterol, triglycerides and glucose were measured in plasma using a Roche Cobas 8000 integrated analyser (Roche Diagnostics GmbH, Mannheim, Germany). LDL(Low-Density Lipoproteins) cholesterol level was estimated using the Friedewald formula. 

### 2.4. Statistics 

Data are presented as the mean ± standard deviation. The statistical analysis was performed using the software Statistical Package for Social Sciences software (SPSS/PC for Windows version 23.0, IBM Corporation, Armonk, NY, USA). Differences in the measured parameters were analyzed by both parametric (paired T-student test) and nonparametric tests (Wilcoxon test for paired data), but only parametric results are reported. We always used a two-tailed test, and *p* < 0.05 was considered statistically significant. We tested for possible confounding of either a period effect or a carry-over effects by the methods reviewed by Senn [20].

## 3. Results 

Nineteen healthy male volunteers, aged 21–32 years (24.6 ± 3.0 years), participated in the study; the average BMI and waist circumference were 22.6 ± 2.6 kg/m^2^ and 84.7 ± 7.9 cm, respectively. No healthy volunteers discontinued the study and none reported any symptoms. Blood pressure, indices of vascular function, along with data on fasting plasma glucose and lipids of the healthy volunteers was collected in the morning of the hemodynamic evaluation, and is presented in Table 1. 

### Effect of the Intervention on Flow Mediated Dilatation and BP

At baseline (morning examination, 1 h before FM), no difference was observed in Flow Mediated Dilatation as well as in the other vascular and metabolic parameters of participants assigned to 1 week of LFD + TP, as compared to LFD-only (Table 1), apart from a slightly lower Stiffness Index in the LFD-only group.

Two hours after the FM (Table 2 and Supplementary Table S1), triglycerides increased significantly in both the LFD and TP + FM, and in the LFD-only + FM groups (*p* < 0.001), instead, plasma total cholesterol and LDL decreased. 

Glucose level increased significantly in the LFD-only + FM group but not in the LFD and TP + FM. 

About BP and vascular parameters, 2 h after the FM, no significant variation was found in Flow Mediated Dilatation. Nevertheless, we observed a significant increase with respect to baseline of the brachial artery diameter (from 4.0 ± 0.4 mm to 4.2 ± 0.4 mm; ∆-diameter 0.2 ± 0.4; *p* = 0.004), a significant decrease in diastolic BP (from 73.3 ± 6.1 mmHg to 70.7 ± 6.8 mmHg; ∆-diastolic BP −2.6± 4.3 mmHg; *p* = 0.017), and in the Stiffness Index (from 6.6 ± 0.6 to 6.4 ± 0.6 m/s; ∆-SI: −0.2 ± 0.4 m/s; *p* = 0.046), exclusively in the LFD and TP + FM group, despite no difference in the comparison of the same vascular and metabolic parameters between LFD and TP + FM and LFD-only + FM (Table S1). 

An increase in heart rate and a decrease in the Reflection Index (*p* < 0.001 for both with respect to baseline) were detected in both groups (LFD and TP + FM and LFD-only + FM).

**Figure 1:**
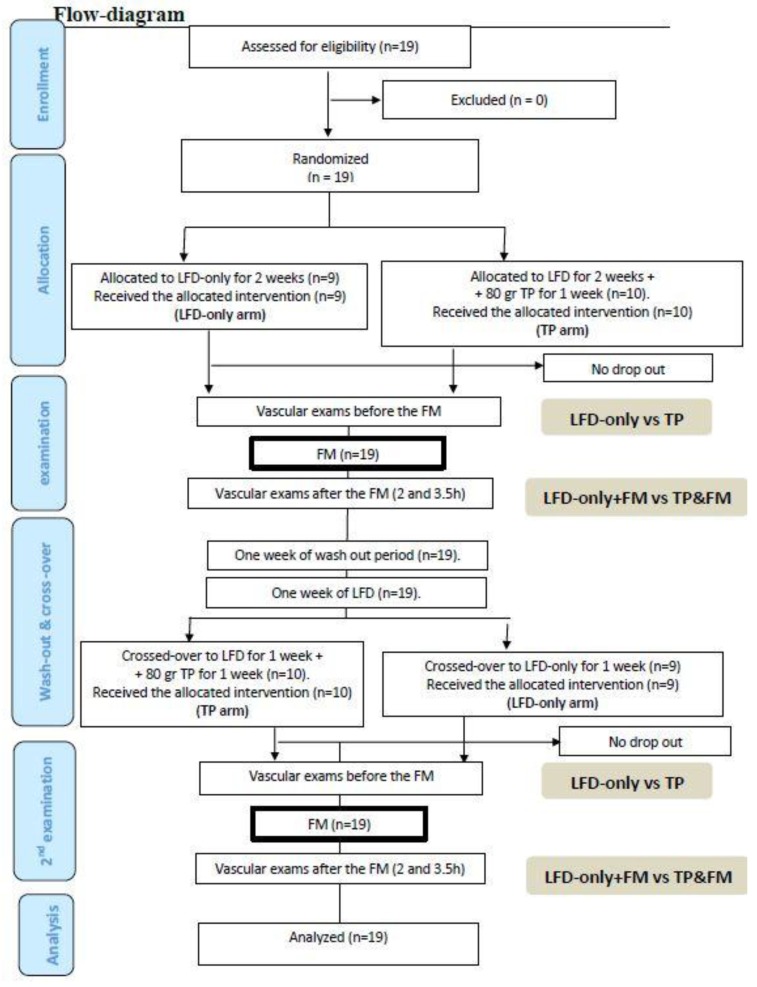
Flow-diagram.

The changes with respect to baseline were not significantly different between the LFD and TP + FM and the LFD-only + FM groups apart from the Stiffness Index: (−0.4 ± 0.8 m/s; *p* = 0.049; Table 3 and Figure 1).

Most of the metabolic changes, occurring at the 2 h point, were maintained at 3.5 h after the meal; however, the Stiffness Index was significantly increased in the LFD-only + FM group (from 6.3 ± 0.5 to 6.7 ± 0.7 m/s; ∆-Stiffness Index: 0.4 ± 0.6 m/s; *p* = 0.023) with a significant difference with respect to the LFD and TP + FM group (−0.6 ± 0.9 m/s; *p* = 0.023).

About Flow Mediated Dilatation, since it is dependent on brachial artery diameter and the latter parameter varied significantly in the LFD and TP + FM group; we repeated the analyses using linear regression to account for the diameter variation obtained no differences with the non-adjusted results presented above.

Nitrate levels in urine, collected during the day of the hemodynamic study, were significantly lower in subjects taking LFD-only + FM with respect to LFD and TP + FM (82.9 ± 37.5 vs. 119.8 ± 80.0 µmoL/mol creatinine, *p* < 0.05).

Repetition of the analyses using nonparametric tests yielded, substantially, the same results (data not shown). 

After adjusting for the period effect, the difference in Stiffness Index between baseline and 2 h in the LFD and TP + FM arm remained borderline significant (*p* = 0.053), as well as the difference between baseline and 3.5 h in the LFD-only + FM group (*p* = 0.02). Even the delta difference between LFD and TP + FM vs LFD-only + FM at 2 h (*p* = 0.057), and at 3.5 h (*p* = 0.02) remained significant. No carry-over effect was detectable (*p* > 0.05 for all the variables tested).

## 4. Discussions

Even if several studies report a reduction in Flow Mediated Dilatation after FM [21,22,23], we did not find a significant worsening of Flow Mediated Dilatation after the FM both in the LFD-only + FM and in the LFD and TP + FM groups. This apparent discrepancy could be due to three types of factors: (i) Related to the FM, (ii) related to the healthy volunteers, or (iii) related to the measure of Flow Mediated Dilatation itself. About factors related to the meal, we know that other studies utilized a higher percentage of fat in the FM. Nevertheless, the FM we used is more adherent to a typical Italian high FM, and despite not having modified Flow Mediated Dilatation, it could modify other important hemodynamic parameters (see below). Second, about factors related to the healthy volunteers, we underline, that in our study, differently from others, they were particularly young and completely free from CV risk factors. Finally, about the Flow Mediated Dilatation technique itself, we know that it exists a high inter and intra-individual variability [24], and this could be responsible for the blurred results we obtained. We tried to standardize the environmental conditions during vascular inspection of the healthy volunteers, according to the recommended protocols, and to put the probe exactly in the same point as in previous examinations of the participant; we also used a stereotactic arm to fix the probe and analyses of images were done by a robust edge detection algorithm that is used to automatically locate the two walls of the vessel with a temporal resolution of 25 frame/s, implemented in a dedicated hardware [25]; finally we tried also to correct the possible bias of difference in the basal diameter by linear regression, but acknowledge the lack of adjustment for the stimulus (shear stress), not available with the current version of our hardware. 

Despite the negative result about the main endpoint, our study shows several interesting findings in secondary end-points. First, hemodynamic changes due to the FM are different in subjects, assuming the LFD-only + FM with respect to volunteers assuming LFD and TP + FM. In particular, in all participants, regardless of the type of FM (LFD-only + FM vs. LFD and TP + FM), we observed, with respect to baseline, an augmented heart rate and a decrease Reflection Index, index of a relative vasodilatation. However, in the TP + FM group, we also detected a decrease in diastolic BP, accompanied by a higher brachial artery diameter and a decrease in Stiffness Index. 

The Stiffness Index also showed a significant decrement with respect to the baseline in the LFD and TP + FM group, as compared with the slight increment in the LFD-only + FM group both at two and 3.5 h after the meal.

Altogether, these results suggest that the acute consumption of TP blunts the effect of a FM on systemic hemodynamics, allowing a healthier vascular state, which is characterized by a more pronounced vasodilatation (decrease in diastolic BP and the Reflection Index, other than an increase in brachial artery diameter) and a better functional elasticity by systemic arteries (decrease in Stiffness Index). The mediator of this effect is likely to be a vasodilator and nitric oxide (NO), despite the lack of the difference observed in Flow Mediated Dilatation, remains as a possible culprit. This hypothesis is also corroborated by the fact that nitrates are higher in the urine of subjects, assuming LFD and TP + FM, even if urinary nitrates can be considered only a row marker of endogenous NO production [26]. Moreover, all types of vegetables, including tomato, are a good source of nitrates that could have beneficial effects on the vascular system, even beyond a specific effect on FMD, together with potassium, which is also relatively abundant in tomato products [27,28]

Indeed, we speculate that the observed hemodynamic beneficial effect of TP could be due to the antioxidant and/or anti-inflammatory effect of lycopene, as detected in other studies [29,30,31], but we did not measure it directly. Thus, we cannot exclude that the beneficial effect of tomato products may be attributed to a sum of the effects of lycopene with other well-known micronutrients such as potassium, nitrates, α-tocopherol, and vitamin C, and newer compounds such as mini-knot proteins [32]. The use of a commercially available tomato product, which cannot help us to unravel this issue, allows a more direct extrapolation of our findings to everyday life.

Additionally, other studies about the effect of tomato on endothelial function as measured by Flow Mediated Dilatation, showed contrasting results, whereas, a two-week period of 70 g of TP supplementation could improve the endothelial function in a group of healthy males and females [33], one week of 70 g of tomato purée, added to a buttered roll, did not show any effect in healthy non-smoking postmenopausal women [34]; indeed, no significant effect in Flow Mediated Dilatation was detectable 3.5 h after a fat meal, despite an improvement in oxidative stress and inflammation in healthy volunteers [31]. Using other techniques to measure endothelial function, notably the reactive hyperemia peripheral arterial tonometry (RH-PAT), Kim et al. found a positive effect on endothelial function and oxidative stress [30].

Nevertheless, a larger clinical trial, involving more than 200 overweight middle-aged volunteers, showed that 12 weeks of a control diet (low in tomato-based foods) was not different to a high-tomato-based diet, or a control diet supplemented with lycopene capsules (10 mg/day) at reducing conventional CVD risk markers, including markers of the endothelial function such as ICAM-1, of oxidation, inflammation, and DVP-Stiffness Index [33]. Other trials using tomato did not measure arterial stiffness [33,34].

About BP, other studies also showed contrasting effects: In some clinical trials lasting at least four weeks, oral supplementation with tomato extract, lycopene or tomato juice sometimes observed significantly decreased BP [30,35], but some others did not [36,37]. The most recent meta-analysis, summarizing all these results, showed a significant effect for systolic, but not diastolic BP [35].

The already mentioned clinical trials about endothelial function, randomizing tomato consumption, but for shorter periods, it did not show any effect in both systolic and diastolic BP [34], or did not present data about BP [31,33].

Our study did not show any significant effect after short-term (one week) supplementation of TP, when fasting, but showed a modest decrease in diastolic BP after the FM, suggesting at least an acute effect when deleterious vascular effects are elicited by an FM. Interestingly, another study comparing two different types of FM, either rich in oleic acid, a monounsaturated fat, or in palmitic acid, saturated fat showed an increase in post-prandial BP, evident for both meals [38] and accompanied by a significant decrease in the Augmentation Index, marker of a reduced velocity of the reflected pulse wave [38].

While many studies have examined the effects of post-prandial glucose variability on cardiovascular outcomes [39,40], relatively few studies have explored the associations of post-prandial BP variations with CV problems [41,42,43].

However, this is conceivable since BP variability in general, either measured during a 24 h ambulatory blood pressure monitoring or from visit to visit, which have been recognized as adjunctive risk factors for CV disease [44].

## 5. Conclusions

In conclusion, despite no effect on Flow Mediated Dilatation, in this randomized cross-over trial, the adding of TP to a FM caused, two h after the meal, some beneficial hemodynamic effects, and in particular, a lower diastolic BP and Stiffness Index with a contemporary increase in urine nitrates. On the light of epidemiological studies, showing a possible positive effect of tomato on CV outcomes and possible antioxidant and vasodilatory effects mediated by lycopene and other micronutrients, we suggest that tomato, a traditional ingredient of a Mediterranean diet, and in particular TP, could exert its beneficial effect by neutralizing deleterious hemodynamic actions by other foods, especially if rich in fat. This information can be important for public health

## Figures and Tables

**Table 1:** Effects of 1 week of tomato paste on blood pressure (BP), vascular exams, and metabolic parameters in healthy young men.

Variable at Baseline	LFD & TP	LFD-Only	Difference LFD & TP vs. LFD-Only	*p*-Value LFD & TP vs. LFD-Only
**SBP (mmHg)**	120.4 ± 7.1	122.2 ± 13.2	−1.8 ± 8.9	0.39
**DBP (mmHg)**	73.3 ± 6.1	72.2 ± 7.2	1.1 ± 5.4	0.37
**HR (bpm)**	64.0 ± 9.5	64.1 ± 13.1	−0.1 ± 10.6	0.98
**Brachial artery diameter (mm)**	4.0 ± 0.4	4.0 ± 0.3	−0.1 ± 0.3	0.53
**FMD (%)**	3.5 ± 1.6	4.4 ± 3.3	−0.9 ± 3.4	0.27
**NMD (%)**	2.6 ± 1.9	3.4 ± 2.6	−0.9 ± 2.6	0.31
**Carotid DC (KPa ^−1^ 10 ^−3^)**	36.4 ± 5.3	35.3 ± 10.3	1.0 ± 9.4	0.49
**SI (m/s)**	6.6 ± 0.6	6.3 ± 0.5	0.3 ± 0.5	0.025
**RI (%)**	71.6 ± 11.2	68.0 ± 14.0	3.6 ± 13.4	0.25
**Total cholesterol (mg/dL)**	154.5 ± 27.2	151.1 ± 26.8	3.4 ± 15.0	0.34
**HDL-cholesterol (mmol/L)**	52.4 ± 15.3	52.0 ± 13.5	0.4 ± 6.2	0.77
**LDL-cholesterol (mmol/L)**	87.4 ± 24.5	84.2 ± 25.8	3.3 ± 12.4	0.51
**Triglycerides (mmol/L)**	78.2 ± 28.0	71.5 ± 30.3	6.7 ± 22.3	0.21
**Glucose (mmol/L)**	86.2 ± 6.3	85.8 ± 4.5	0.5 ± 7.2	0.78

LFD, Low Fiber Diet; TP, Tomato Paste; SBP, Systolic Blood Pressure; DBP, Diastolic Blood Pressure; HR, Heart Rate; FMD, Flow Mediated Dilatation; NMD, nitroglycerin mediated dilatation; DC, distensibility; SI, Stiffness Index; RI, Reflection Index, HDL, High density Lipoproteins; and LDL, Low density Lipoproteins.

**Table 2:** Difference with respect to baseline of BP, vascular exams and metabolic parameters after 2 and 3.5 h from a fat meal ± tomato paste (LFD and TP + FM vs. LFD-only + FM ) in healthy young men.

Variable at Baseline	Difference LFD & TP + FM (2 h) vs. Baseline	*p*-Value LFD & TP + FM (2 h) vs. Baseline	Difference LFD-Only + FM (2 h) vs. Baseline	*p*-Value LFD-Only + FM (2 h) vs. Baseline	Difference LFD & TP + FM (3.5 h) vs. Baseline	*p*-Value LFD & TP + FM (3.5 h) vs. Baseline	Difference LFD-Only + FM (3.5 h) vs. Baseline	*p*-Value LFD-Only + FM (3.5 h) vs. Baseline
**SBP (mmHg)**	1.5 ± 6.9	0.35	0.2 ± 8.2	0.93	0.8 ± 7.8	0.67	−1.2 ± 9.0	0.56
**DBP (mmHg)**	−2.6 ± 4.3	0.017	−0.3 ± 6.3	0.82	−0.3 ± 6.4	0.82	−1.3 ± 5.1	0.30
**HR (bpm)**	4.1 ± 4.8	0.001	4.5 ± 6.9	0.011	2.4 ± 6.2	0.11	0.3 ± 9.9	0.90
**Brachial artery diameter (mm)**	0.2 ± 0.2	0.004	0.1 ± 0.3	0.26	0.1 ± 0.6	0.62	0.1 ± 0.3	0.28
**FMD (%)**	0.7 ± 3.0	0.32	−1.0 ± 3.5	0.23	0.6 ± 3.0	0.41	0.1 ± 5.0	0.95
**NMD (%)**	1.4 ± 3.1	0.16	0.6 ± 1.7	0.36	n.p.	n.p.	n.p.	n.p.
**Carotid DC (KPa ^−1^ 10 ^−3^)**	−0.2 ± 5.7	0.91	3.4 ± 10.5	0.17	0.7 ± 4.5	0.50	2.2 ± 8.7	0.29
**SI (m/s)**	−0.2 ± 0.4	0.046	0.2 ± 0.6	0.17	−0.2 ± 0.5	0.09	0.4 ± 0.6	0.023
**RI (%)**	−11 ± 7.4	<0.001	−6.6 ± 7.2	0.006	−5.7 ± 9.5	0.019	1.3 ± 9.8	0.56
**Total cholesterol (mg/dL)**	−3.3 ± 10.7	0.19	−2.8 ± 7.3	0.11	−2.0 ± 8.4	0.32	−3.3 ± 10.2	0.17
**HDL-cholesterol (mmol/L)**	−3.4 ± 4.1	0.002	−3.7 ± 3.3	<0.001	−4.3 ± 3.2	<0.001	−4.8 ± 3.8	<0.001
**LDL-cholesterol (mmol/L)**	−13.0 ± 10.0	<0.001	−10.9 ± 8.5	<0.001	−10.2 ± 8.9	<0.001	−10.1 ± 12.4	0.002
**Triglycerides (mmol/L)**	72.9 ± 40.8	<0.001	69.4 ± 34.3	<0.001	65.9 ± 45.0	<0.001	66.8 ± 44.6	<0.001
**Glucose (mmol/L)**	6.6 ± 20.4	0.18	9.9 ± 12.8	0.003	2.3 ± 8.8	0.28	5.2 ± 5.4	<0.001

FM, Fat Meal; TP, Tomato Paste; SBP, Systolic Blood Pressure; DBP, Diastolic Blood Pressure; HR, Heart Rate; FMD, Flow Mediated Dilatation; NMD, nitroglycerin mediated dilatation; DC, distensibility; SI, Stiffness Index; RI, Reflection Index, HDL, High density Lipoproteins; and LDL, Low density Lipoproteins.

**Table 3:** Difference between volunteers who consumed a LFD and TP + FM with respect to LFD-only + FM in the changes with respect to baseline of BP, vascular exams and metabolic parameters.

Variable at Baseline	Difference between Δ-LFD & TP + FM vs. Δ-LFD-Only +FM (2 h)	*p*-Value Δ-LFD & TP + FM vs. Δ-LFD-Only + FM (2 h)	Difference between Δ-LFD & TP + FM vs. Δ-LFD-Only + FM (3.5 h)	*p*-Value Δ-LFD & TP + FM vs. Δ-LFD-Only + FM (3.5 h)
**SBP (mmHg)**	1.4 ± 11.6	0.61	0.6 ± 12.4	0.83
**DBP (mmHg)**	−2.29 ± 7.2	0.18	0.9 ± 7.0	0.58
**HR (bpm)**	−0.3 ± 7.0	0.83	2.1 ± 9.3	0.34
**Brachial artery diameter (mm)**	0.1 ± 0.4	0.23	0.0 ± 0.6	0.90
**FMD (%)**	1.7 ± 4.1	0.085	0.5 ± 5.7	0.69
**NMD (%)**	1.6 ± 3.0	0.19	n.p.	n.p.
**Carotid DC (KPa ^−1^ 10 ^−3^)**	−3.6 ± 11.8	0.20	−1.5 ± 7.6	0.41
**SI (m/s)**	−0.4 ± 0.8	0.048	−0.6 ± 0.9	0.022
**RI (%)**	−4.5 ± 13.3	0.16	−4.3 ± 15.4	0.23
**Total cholesterol (mg/dL)**	−0.1 ± 14.7	0.99	1.3 ± 14.9	0.70
**HDL-cholesterol (mmol/L)**	0.3 ± 5.4	0.80	0.5 ± 5.7	0.72
**LDL-cholesterol (mmol/L)**	−2.1 ± 11.8	0.46	−0.1 ± 9.5	0.97
**Triglycerides (mmol/L)**	3.5 ± 41.4	0.72	−0.8 ± 28.3	0.90
**Glucose (mmol/L)**	−3.4 ± 22.9	0.53	−2.9 ± 10.6	0.24

FM, Fat Meal; TP, Tomato Paste; SBP, Systolic Blood Pressure; DBP, Diastolic Blood Pressure; HR, Heart Rate; FMD, Flow Mediated Dilatation; NMD, nitroglycerin mediated dilatation; DC, distensibility; SI, Stiffness Index; RI, Reflection Index, HDL, High density Lipoproteins; and LDL, Low density Lipoproteins.

## Supplementary Materials

The following are available online at http://www.mdpi.com/2072-6643/10/9/1310/s1, Table S1: Effect (at 2 and 3.5 h) of a fat meal ± tomato paste (FM ± TP) on BP, vascular exams and metabolic parameters in healthy, young men.

## Abbreviations

BP	Blood pressure
FM	Fatty Meal
IMT	Intima Media Thickness
LFD	Low-Fiber Diet
NO	Nitric Oxide
TP	tomato paste
LFD	low fiber diet
FM	Fatty meal
