# Supplementary material for: Positive Effects of Tomato Paste on Vascular Function After a Fat Meal in Male Healthy Subjects
Source: Nutrients. 2018 Sep 15;10(9):1310. doi: 10.3390/nu10091310 (PMC6163719; doi:10.3390/nu10091310)
Supplement: Supplementary file 1 [file nutrients-10-01310-s001.pdf]

Supplementary Materials: Positive effects of Tomato Paste on vascular function after a Fat Meal in Healthy Subjects.

Andrea Dalbeni [1,*], Davide Treggiari [2], Angela Tagetti [1], Michele Bevilaqua [1], Sara Bonafini [1], Martina Montagnana [3], Giuliana Scaturro [1], Pietro Minuz [1] and Cristiano Fava[1]

Table S1. Effect (at 2 and 3.5 hours) of a fat meal ± tomato paste (FM±TP) on BP, vascular exams and metabolic parameters in healthy, young men.

Variable at baseline	LFD&TP+FM (2h)	LFD-only+FM (2h)	Difference LFD&TP+FM (2h) vs. LFD-only+FM (2h)	P-value LFD&TP+FM (2h) vs. LF-only+FM (2h)	LFD&TP+FM (3.5h)	LFD-only+FM (3.5h)	Difference LFD&TP+FM (3.5h) vs. LFD-only+FM (2h)	P-value LFD&TP+FM (3.5h) vs. LFD-nly+FM (2h)
**SBP (mmHg)**	121.9 ± 6.6	122.4±8.8	-0.4±9.0	0.84	121.2±8.9	121.0±9.6	0.2±9.3	0.92
**DBP (mmHg)**	70.7 ±6.8	71.8±6.8	-1.2±5.8	0.39	72.9±8.5	70.9±7.2	2.1±8.0	0.28
**HR (bpm)**	68.2±9.2	68.6±11.4	-0.4±6.9	0.80	66.4±11.2	64.4±9.3	2.0±8.4	0.31
**Brachial artery diameter (mm)**	4.2±0.4	4.1±0.4	0.1±0.4	0.39	4.1±0.7	4.1±0.4	-0.0±0.6	0.84
**FMD (%)**	4.2±2.6	3.4±2.7	0.8±3.1	0.27	4.1±2.0	4.5±3.5	-0.4±3.7	0.67
**NMD (%)**	4.1±2.2	3.4±2.2	0.6±3.2	0.58	n.p.	n.p.	n.p.	n.p.
**Carotid DC ($KPa^{-1}10^{-3}$)**	36.2±5.3	38.8±7.0	-2.6±6.1	0.08	37.1±4.9	37.5±7.1	-0.4±7.1	0.75
**SI (m/sec)**	6.4±0.6	6.5±0.8	-0.1±0.6	0.52	6.4±0.6	6.7±0.7	-0.2±0.8	0.20
**RI (%)**	60.6±10.9	61.4±12.1	0.9±11.3	0.76	66.0±11.6	66.7±10.9	-0.7±10.8	0.76
**Total cholesterol (mg/dL)**	151.2±25.4	148.3±28.3	2.8±18.3	0.51	152.5±25.5	147.8±25.2	4.6±15.8	0.21
**HDL-cholesterol (mmol/L)**	49.0±14.2	48.3±12.9	0.7±5.1	0.54	48.1±14.5	47.2±14.0	0.9±3.8	0.33
**LDL-cholesterol (mmol/L)**	74.5±24.7	73.3±26.9	1.1±13.2	0.71	77.2±24.9	74.1±23.2	3.1±10.4	0.21
**Triglycerides (mmol/L)**	151.0±59.3	140.9±49.8	10.2±45.4	0.34	144.1±64.5	138.3±69.4	5.8±39.3	0.52
**Glucose (mmol/L)**	92.8±21	95.7±12.4	-2.9±22.1	0.57	88.6±8.4	91.0±6.5	-2.4±9.4	0.27

FM, Fat Meal; TP, Tomato Paste; SBP, Systolic Blood Pressure; DBP, Diastolic Blood Pressure; HR, Heart Rate; FMD, Flow Mediated Dilatation; NMD, nitroglycerin mediated dilatation; DC, distensibility; SI, Stiffness Index; RI, Reflection Index, HDL, High density Lipoproteins; LDL, Low density Lipoproteins; n.p., not performed.
